# Effect of lite touch on the anxiety of low-risk pregnant women in the latent phase of childbirth: a randomized controlled trial

**DOI:** 10.3389/fpsyg.2024.1304274

**Published:** 2024-02-05

**Authors:** Wenqian Yang, Yonghong Wang, Chingyuan Ko, Xiaoyu Niu, Yan Huang, Biru Luo, Guoyu Wang, Jingjing He, Huafeng Li

**Affiliations:** ^1^Department of Obstetrics Nursing, West China Second University Hospital, Sichuan University, Chengdu, Sichuan, China; ^2^Key Laboratory of Birth Defects and Related Diseases of Women and Children (Sichuan University), Ministry of Education, Chengdu, Sichuan, China; ^3^Department of Nursing, West China Second University Hospital, Sichuan University, Chengdu, Sichuan, China; ^4^Department of Gynecology, West China Second University Hospital, Sichuan University, Chengdu, Sichuan, China; ^5^Department of Anesthesiology, West China Second University Hospital, Sichuan University, Chengdu, Sichuan, China

**Keywords:** anxiety, catecholamines, hydrocortisone, pregnancy, touch

## Abstract

**Introduction:**

Women with perinatal anxiety have reduced coping capacity during labor, which affects labor progress and increases the likelihood of a cesarean section. Several non-pharmacological interventions for anxiety during childbirth are available. This study used the “lite touch” method, a non-pharmacological intervention based on physiological responses and obstetric clinical experience in women. We aimed to evaluate whether lite touch could relieve perinatal anxiety and investigate the effect of light skin stroking on the maternal hormones, catecholamine, and cortisol.

**Methods:**

This randomized clinical trial involved women with low-risk singleton pregnancies at full term or near term. Eligible pregnant women who were latent and did not undergo epidural anesthesia were randomized into two groups. Participants in the intervention group underwent routine prenatal care, including lite touch, whereas the control group underwent routine prenatal care alone. Demographic data were collected through a questionnaire. Labor anxiety was assessed using the State Anxiety Inventory, and saliva was collected before and after the intervention. Changes in saliva cortisol and catecholamine levels were analyzed using a double-antibody sandwich enzyme-linked immunosorbent assay.

**Results:**

In total, 83 participants were included, with 43 and 40 in the intervention and control groups, respectively. In the intervention group, pre-intervention anxiety scores were significantly lower (*p* < 0.01) than post-intervention anxiety scores, whereas the control group showed no difference in anxiety scores before and after intervention (*p* > 0.05). Cortisol and catecholamine levels in saliva were significantly lower in the intervention group than in the control group after the intervention (*p* < 0.01).

**Discussion:**

Lite touch can reduce the latent anxiety state of low-risk pregnant women, thereby maintaining *in vivo* stability and facilitating labor.

**Clinical trial registration:**

https://www.chictr.org.cn/aboutEN.html, ChiCTR2300070905, Retrospectively Registered Date: April 26, 2023.

## 1 Introduction

Pregnancy involves physical and psychological changes that may increase the risk of mental health issues, especially in developing countries (Fisher et al., 2012). Perinatal anxiety is a burden to pregnant women worldwide, particularly to those in low and middle-income countries (Nielsen-Scott et al., 2022). The prevalence of prenatal anxiety in China is 17.4% (Yang et al., 2023). Negative thinking in the perinatal period can adversely impact the health of the woman’s child. Moreover, women with perinatal anxiety tend to opt for cesarean section, increasing the prevalence of cesarean sections for non-medical reasons at term (Rubertsson et al., 2014). The degree of tokophobia in women increases with anxiety, leading to reduced coping capacity of women during labor, affecting labor progress (George et al., 2013). Moreover, anxiety adversely affects maternal–infant bonding (Benarous et al., 2023) and is closely related to post-partum depression (Kaydırak et al., 2022). Researchers have explored several non-pharmacological interventions to reduce perinatal anxiety among pregnant women. Ebrahimian et al. (2022) found that chewing gum can reduce anxiety levels in the first stage of labor. Furthermore, playing music can reduce anxiety levels during labor (Lin et al., 2019; Buglione et al., 2020). Massages to relieve anxiety can also increase maternal satisfaction during labor (Akköz Çevik and Karaduman, 2020; Shahbazzadegan and Nikjou, 2022). Aromatherapy, as a complementary and alternative modality, can relieve maternal anxiety during labor (Hamdamian et al., 2018; Tabatabaeichehr and Mortazavi, 2020). Overall, these non-pharmacological interventions are widely used in developed countries or advanced hospitals, which have effectively relieved perinatal anxiety in clinical practice. However, most of these interventions require professional training, which is a barrier to their popularization in low- and middle-income countries. Hence, there remains a lack of non-pharmaceutical interventions suitable for developing countries (Dennis et al., 2017).

Consequently, it is necessary to develop an easy to implement, low-cost, and evidence-based non-pharmaceutical intervention that can be utilized in developing countries or hospitals with underdeveloped medical skills. This type of non-pharmacological intervention may improve childbirth quality and reduce hospital costs.

This study assessed the lite touch method, which was first developed by Constance Palinsky. This method can reduce discomfort and increase bodily relaxation by stimulating the skin surface (Constance and Larry, 2008). Subsequently, the lite touch method has been improved with obstetric clinical experience. The method involves using the cotton wool of disposable cotton swabs to lightly stroke a specific area on the skin of pregnant women in labor to elicit a “ticklish” sensation. We aimed to explore whether lite touch could relieve perinatal anxiety and further investigated the effect of lite touch on maternal hormones, catecholamine, and cortisol. This study had two aspects: (1) The intervention used in this study is an inexpensive, easily accessible, and non-invasive non-pharmaceutical intervention based on midwives’ clinical practice experience; and (2) We explored the changes in the internal environment of pregnant women due to the intervention from a physiological perspective.

## 2 Methods

### 2.1 Study design and procedure

This study was a randomized controlled trial. Participants were pregnant women with perinatal health care cards who underwent periodic maternity check-ups at the West China Second University Hospital of Sichuan University and were admitted to the delivery room during labor between March and May 2023. The inclusion criteria were as follows: (1) singleton, primiparous, or perinatal pregnancies; (2) ability to undergo vaginal birth after assessment by an obstetrician; at this hospital, pregnant women undergo comprehensive evaluation by obstetricians using specialized data, and obstetricians then provide recommendations on the suitability of vaginal delivery; (3) admission to the delivery room without an epidural when the participant is officially in labor; and (4) willingness to cooperate. The exclusion criteria were as follows: (1) psychiatric or speech disorders; (2) major medical and surgical comorbidities; (3) conversion to cesarean delivery midway through the study; and (4) discontinuing the intervention, lite touch, for any reason during the study.

Based on the random concealment of allocation principle, a sequence of random numbers was generated using Excel, sufficient according to the sample size. The numbers were put in sealed opaque envelopes and handed to midwives who did not participate in the study. After participants entered the delivery room, the researchers asked the midwife to divide them into experimental (odd numbers) or control groups (even numbers) using the generated random numbers. The participants were blinded to the intervention received; the investigators, however, were aware of the participant grouping. Statisticians who did not participate in this study conducted the data analysis.

The sample size was calculated using the formula for comparing the two means on the Power and Sample Size online calculator webpage. The State Anxiety Inventory (SAI) mean score was 48.22 in the control group and 43.22 in the intervention group, with a standard deviation of 8.39, k = $n_{A}$ / $n_{B}$ =1, 1−β = 0.80, and α = 5%. Each group had 45 participants, considering a 10% loss to the follow-up rate. The final sample size per group was 50 participants.

$$n_{A}=kn_{B}\;\mathrm{and}\;n_{B}=\left(1+\frac{1}{k}\right)\left(\sigma \frac{z_{1-\alpha /2}+z_{1-\beta }}{\mu_{A}-\mu_{B}}\right)$$

All procedures performed in studies involving human participants were in accordance with the ethical standards of the institutional and national research committee and with the 1964 Helsinki Declaration and its later amendments. The study was approved by the Medical Ethics Committee of the Second West China Hospital of Sichuan University (Approval Number: 2022220). Written informed consent was obtained from all patients; all participants were informed that they could withdraw at any point during the study.

### 2.2 Participants

In total, 83 participants were recruited; the intervention and control groups had 43 and 40 participants, respectively. Participants’ demographic information included age, education, profession, body mass index (BMI), primipara or multipara status, and whether or not the pregnancy was full-term. Based on the detailed data compiled, there were no significant differences between the intervention and control groups (Table 1).

**Table 1:** Comparison of the demographic characteristics of the participants in the intervention and control groups.

Demographic characteristics		Intervention group (*n* = 43)	Control group (*n* = 40)	Statistical coefficient	*p* value
Age (years)		29.47 ± 2.87	29.9 ± 3.19	−0.65	0.52^a^
Education	High school and below	3 (7.0%)	2 (5.0%)	−0.36	0.72^b^
	Diploma	11 (25.6%)	14 (35.0%)		
	Bachelor’s degree	22 (51.2%)	17 (39.5%)		
	Master’s degree and above	7 (16.3%)	7 (17.5%)		
Profession	Professional technical employee	7 (16.3%)	4 (10.0%)	1.70	0.79^c^
	Enterprises	2 (4.7%)	1 (2.3%)		
	General employee	27 (62.8%)	30 (75.0%)		
	Temporarily unemployed	2 (4.7%)	2 (5.0%)		
	Other	5 (11.6%)	3 (7.5%)		
BMI (kg/m^2^) before labor	<18.5	12 (27.9%)	6 (15.0%)	−1.04	0.30^b^
	18.5–24.9	28 (65.1%)	32 (80.0%)		
	25–29.9	1 (2.3%)	2 (5.0%)		
	≥30	2 (4.7%)	0 (0%)		
Para	Primipara	40 (93.0%)	32 (80.0%)	3.06	0.08 ^c^
	Multipara	3 (7.0%)	8 (20.0%)		
Whether it is full-term or not	Yes	43 (100%)	39 (97.5%)		0.48^*^
	No	0 (0%)	1 (2.3%)		

^a^*t*-value, ^b^*Z*-value, ^c^*X*^2^ value, and ^*^Fisher’s exact test.

### 2.3 Interventions

Eligible pregnant women were admitted to the delivery rooms and randomly assigned to treatment or control groups. Pregnant women in the control group underwent routine delivery care; after admission to the delivery rooms, they assumed a comfortable position and underwent a vaginal examination and electronic fetal monitoring for 30 min. During the contraction process, the midwives facilitated the Lamaze breathing technique to relieve contraction pain. After the 30-min electronic fetal monitoring, epidural anesthesia was administered after evaluation by the anesthesiologist. At this hospital, 90% of women opt for epidural analgesia during childbirth, whereas the remaining 10% refrain from using epidural analgesia due to rapid progression of the labor process, leading to a relatively short duration of childbirth.

**Figure 1:**
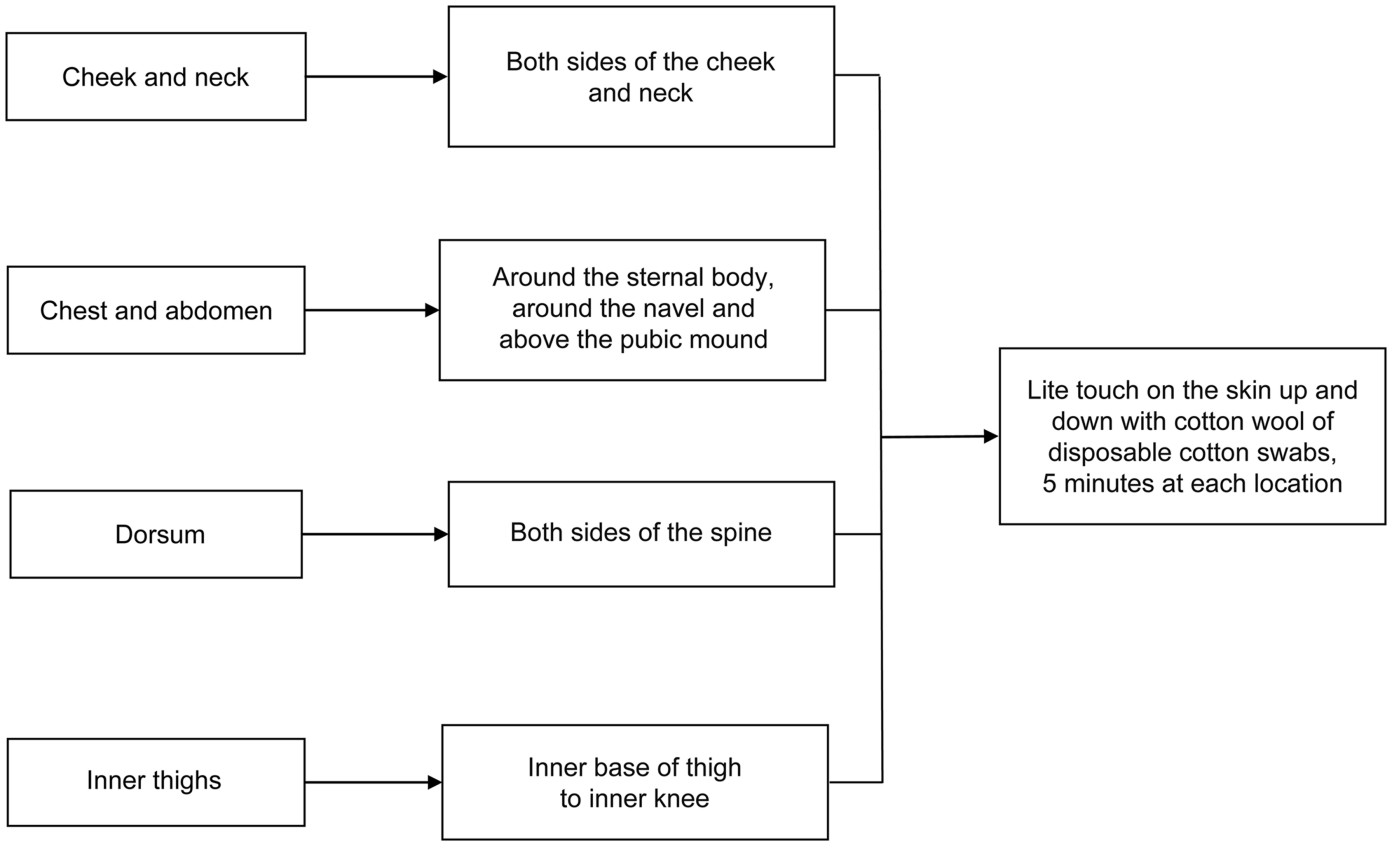
Flowchart of intervention procedure.

**Figure 2:**
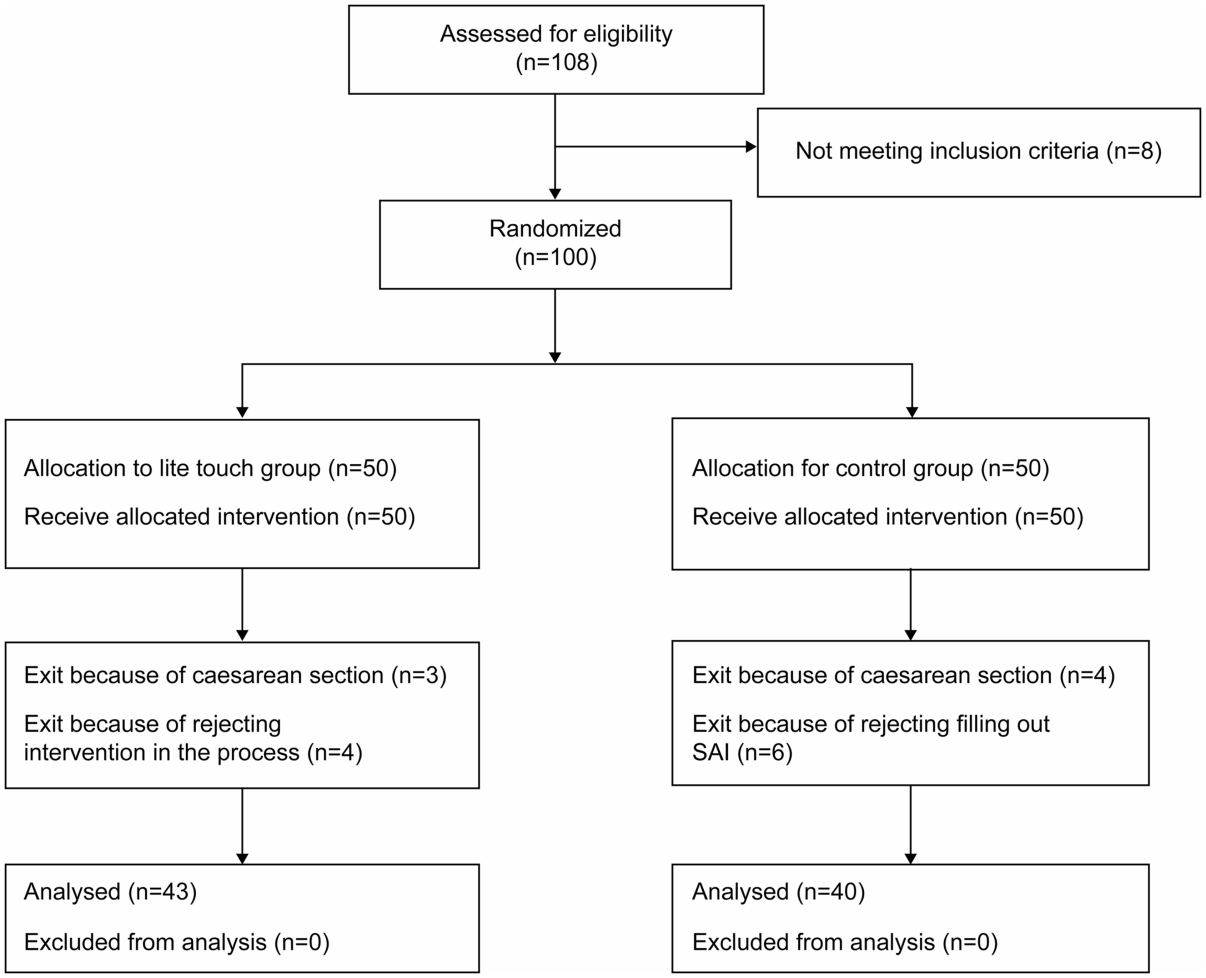
Trial flowchart. SAI, State anxiety inventory.

In contrast, the treatment group underwent lite touch in addition to routine normal delivery care. To prevent nosocomial infection, disposable cotton swabs were used for lite touch. During the 30-min electronic fetal monitoring, the cotton wool of disposable cotton swabs was used to lightly touch specific areas on the skin of pregnant women in labor to elicit a “ticklish” sensation. The specific areas included the cheek, neck, chest, abdomen, dorsum, and inner thighs. Each area was touched for 5 min, with the intervention lasting for approximately 20 min. The touching strength was strong enough that they could feel the cotton wool. The intervention procedure flow is illustrated in Figure 1. After electronic fetal monitoring and evaluation by an anesthesiologist, an epidural anesthesia was administered (Figure 2).

### 2.4 Outcome measures

The outcome measures in this study included anxiety and saliva hormone levels. Anxiety was evaluated Studies have demonstrated that a pregnant women’s anxiety is state anxiety (Delgado et al., 2016); hence, this study evaluated the extent of perinatal anxiety in pregnant women using the Chinese version of the State Anxiety Inventory (SAI), a subscale of the Chinese version of the State–Trait Anxiety Inventory developed by Spielberger in 1983 (Ferreira and Murray, 1983). The Chinese version of the SAI has 20 items, good reliability, and is commonly used in the medical field and. The score was recorded using the four-point Likert scale to reflect nervous and anxious feelings in specific periods, with a total score of 20–80. A higher score correlated with more severe anxiety symptoms. Cronbach’s *α* value of this study was 0.89.

Saliva hormone levels analyzed were cortisol and catecholamine.

### 2.5 Data collection

All data were collected for all participants between March and May 2023. Saliva was collected and SAI score determined before and after the intervention by a clinician blinded to the study.

To minimize the effects of oral environment on hormone measurement, each participant was required to rinse their mouth with drinking water prior to saliva sample collection. As hormone production may vary throughout the day, saliva samples were collected between 9:00 am to 12:00 pm every day. The collected saliva was stored in specimen tubes labeled with name, hospitalization number, and time (before/after). The tubes were placed in a liquid nitrogen tank and then collectively sent to the specimen testing office. The saliva samples were analyzed using a double-antibody sandwich enzyme-linked immunosorbent assay. The enzyme-linked immunosorbent assay kit was provided by QUANZHOU RUIXIN Biotechnology Co., Ltd. (Fujian, China). Test instruments included a RT-6100 absorbance microplate reader (Rayto, Shenzhen, China), a Finnpipette (Thermo Fisher Scientific, Massachusetts, United States), an electric heating constant temperature incubator (Yiheng Sujing Scientific, Wuhan, China), a 988 washboard machine (Tianshi Tianxing Technology, Beijing, China), a RT-6100450 nm wavelength Enzyme-linked immunosorbent assay (Rayto, Shenzhen, China), a TG16W Micro high-speed centrifuge (Xiangzhi Centrifuge Instrument, Changsha, China), a TGL16M desktop high-speed freezing centrifuge (Luxiangyi Centrifuge Instrument, Shanghai, China), an HT-111B Constant temperature shaking table (Hetian Scientific Instrument, Shanghai, China), an AE1204 electronic Analytical balance (Liangping Instrument, Shanghai, China), an XB220A electronic Analytical balance (PRECISA, Zurich, Swiss), a 81-2 constant temperature Magnetic stirrer (Meiyingpu Instrument Manufacturing, Shanghai, China), and a JY98-IIIN EasyWell cell disruption instrument (Rongyan Instrument, Shanghai, China).

### 2.6 Statistical analysis

Statistical analyses were performed using the statistical package: SPSS version 25.0 statistical analysis software (IBM Corporation, Armonk, NY, United States). Data were described as means and standard deviations, medians and interquartile ranges, or quantities and percentages. Independent *t*-test, rank sum test, Chi–squared test, and Fisher’s exact test were used to explore the differences between the two groups at baseline. As the SAI scores of the two groups had a normal distribution, a paired *t*-test was used to explore the difference between the SAI scores of the two groups before and after the intervention, and an independent *t*-test was used to explore the difference between the two SAI groups. As physiological data had a non-normal distribution, the Mann–Whitney *U* test was used to investigate the between-group differences in cortisol and catecholamine levels in saliva samples and the differences in the amount of increased cortisol and catecholamine in the two groups. Statistical significance was set at *p* < 0.05.

## 3 Results

The study flow is illustrated in Figure 2.

There were no significant differences in SAI scores (Table 2) and salivary cortisol and catecholamine levels between the intervention and control groups (Tables 3, 4) before the intervention (all *p* > 0.05). The SAI scores before the intervention were significantly higher than those after the intervention in the intervention group (*p* < 0.01). No significant difference was noted in the SAI scores in the control group (*p* > 0.05, Table 2). However, salivary cortisol and catecholamine levels after the intervention were significantly lower in the intervention group than in the control group (all *p* < 0.01, Tables 3, 4).

**Table 2:** Comparison of SAI scores in the intervention and control groups.

	SAI scores before intervention	SAI scores after intervention	*T* value	*p* value
Intervention group (*n* = 43)	50.02 ± 9.44	44.60 ± 9.52	4.26	<0.001^a^
Control group (*n* = 40)	46.3 ± 10.29	47.45 ± 11.36	−0.98	0.34^a^
*T* value	1.72	−1.24		
*p* value	0.09^b^	0.22^b^		

SAI, State anxiety inventory. ^a^represents paired sample *t*-test. ^b^represents independent sample *t*-test.

**Table 3:** Comparison of cortisol in saliva in the intervention and control groups.

	Cortisol level before intervention (nmol/L)	Cortisol level after intervention (nmol/L)	Difference in cortisol content (nmol/L)
Intervention group (*n* = 43)	183.33 (158–203.67)	203.07 (178.90–226.15)	24.569 (−3.92–42.58)
Control group (*n* = 40)	173.93 (156.20–208.09)	238.71 (222.99–284.17)	62.176 (28.52–111.11)
*Z* value	−0.75	−5.20	−4.49
*p* value	0.45	<0.001	<0.001

Difference in cortisol content = cortisol level after intervention − cortisol level before intervention.

**Table 4:** Comparison of catecholamine in saliva in the intervention and control groups.

	Catecholamine level before intervention (ng/mL)	Catecholamine level after intervention (ng/mL)	Difference in catecholamine content(ng/L)
Intervention group (*n* = 43)	138.98 (126.64–151.63)	149.32 (138.10–167.51)	12.73 (3.95–32.12)
Control group (*n* = 40)	138.93 (115.50–155.06)	168.54 (155.73–197.65)	30.66 (8.84–62.96)
*Z* value	−0.292	−4.393	−3.15
*p* value	0.77	<0.001	0.002

Difference in catecholamine levels = Catecholamine level after intervention − Catecholamine level before intervention.

## 4 Discussion

Anxiety causes autonomic dysfunction, leading to uncoordinated contractions, thereby prolonging the duration of labor, and increasing the risk of adverse birth outcomes (Glover, 2014; Tzeng et al., 2017). In this study, post-intervention anxiety scores were significantly lower than pre-intervention anxiety scores in pregnant women in the intervention group (*p* < 0.001). In contrast, no significant differences were observed between the pre- and post-intervention in the control group (*p* > 0.05). These findings indicate that lite touch alleviated latent anxiety in pregnant women.

Currently, there are no studies on the effect of lite touch on perinatal anxiety. The skin of the body part swept by the cotton wool during the lite touch intervention is thin and more sensitive to external stimuli. The cotton wool is used to stimulate specific sensitive areas, causing a brief “ticklish” sensation in the pregnant woman, such as someone gently tickling (knismesis) the pregnant woman. Proelss et al. (2022). found that tickling multiple parts of the body induces pleasure. Kaufmann et al. (2022) found that tickling of rats evoked somatosensory cortex activity while inhibiting anxiety. However, there have been no studies on the effect of tickling on pregnant women. Based on reciprocal inhibition reported by Wolpe et al. (Feinstein et al., 2018), lite touch is like touching. It acts on the skin surface, stimulating afferent impulses from the skin’s touch receptors and integrating them into the hypothalamus, producing a sense of pleasure, comfort, and an anxiolytic effect. In our study, two patients refused the intervention because the midwife was not allowed to touch them after entering the delivery room, and another two patients refused the intervention due to rapid progression of the labor process. At the beginning of the intervention, the cervix opened quickly, and the patient directly entered the second stage.

When the body is under stress, the hypothalamic–pituitary–adrenal axis is activated, and adrenocorticotropic hormones are elevated, thereby stimulating the release of glucocorticoids from the adrenal cortex (de Quervain et al., 2017). Cortisol is the main glucocorticoid regulating the metabolism and utilization of proteins, carbohydrates, fats, and physiological and psychological stress. Cortisol is released in the body under stress (Haj-Yahia et al., 2020), and is therefore a stress biomarker (Benfield et al., 2014). Higher anxiety results in higher stress, thereby resulting in higher cortisol levels in the body (Chojnowska et al., 2021; Chronister et al., 2021). According to Miller et al. (2019), during labor without any intervention, more intense contractions lead to a more stressful birth, with higher cortisol levels. In this study, we intervened during the latent phase and before the epidural was administered. We alleviated tokophobia before the epidural administration by applying lite touch to the skin surface, decreasing labor stress. Our results showed that the difference in the saliva cortisol level of pregnant women in the intervention group was lower than that of the control group (*p <* 0.01). This indicates that the rate of increase in the anxiety level of pregnant women in the intervention group was lower than that of the control group, further demonstrating that lite touch can relieve stress and anxiety during labor.

The sympathetic nervous system is activated under stress, causing an increase in the secretion of catecholamines in the body (Jung et al., 2019). Labor is also a state of stress. Increased catecholamine secretion strengthens myocardial contraction and increases heart rate, heartbeat, and systolic blood pressure (Fernstrom and Fernstrom, 2007). Increased heart and breathing rates cause hyperventilation and reduce blood supply to the brain. This results in a lack of oxygen and increases anxiety in pregnant women, which may lead to reduced oxygen supply to the placenta and affect the state of the fetus in the uterus. In addition, catecholamines divert blood to major muscle groups in the body. This reduces circulation to the uterus and placenta, affects the fetal blood supply, and increases the risk of fetal intrauterine distress (Shirai, 1992; Andrade Santos et al., 2022). However, in this study, stimulating sensitive areas of the skin reduced labor anxiety and tokophobia, alleviated stress response, and reduced catecholamine levels in the body, thereby stabilizing the intrauterine environment of the fetus.

Nonetheless, this study has some limitations. First, it was a single-center study conducted in a tertiary care maternal-fetal hospital in southwest China, with a small sample size and evaluation index, which may have limited its generalizability. Second, the study exclusively focused on low-risk singleton pregnancies at full term or near term, leaving the effect of light skin stroking during vaginal delivery in medium- and high-risk pregnancies unknown. The duration of intervention in this study was limited to the latent phase and before epidural anesthesia administration; therefore, the effect on active labor periods remains unknown. Third, this study represents our initial exploration of lite touch, wherein we did not include any outcome indicators associated with delivery progress. While we recorded whether women were full term during data collection, specific pregnancy week details were not documented. In future research, we aim to enhance data collection rigorously by including more comprehensive data. Further, we also plan to incorporate additional indicators to evaluate the promoting effect of lite touch on childbirth. Therefore, future studies should consider multi-center, large-sample research designs, and abundant evaluation metrics to further demonstrate the effectiveness of this convenient, low-cost, readily available, non-pharmacological, non-invasive intervention on pregnant women in labor.

In conclusion, lite touch during the latent phase in pregnant women reduces labor anxiety and cortisol and catecholamine levels in low-risk pregnant women. Therefore, we recommend lite touch as an intervention during the latent phase in low-risk pregnancies to alleviate maternal anxiety, maintain the stability of the internal environment, and facilitate labor.

## Data availability statement

The datasets presented in this study can be found in online repositories. The names of the repository/repositories and accession number(s) can be found here: Clinical Trial Management Public Platform, http://www.medresman.org.cn/login.aspx (Enter using Public Access).

## Ethics statement

The studies involving humans were approved by Medical Ethics Committee of the Second West China Hospital of Sichuan University. The studies were conducted in accordance with the local legislation and institutional requirements. The participants provided their written informed consent to participate in this study.

## Author contributions

WY: Writing – original draft. YW: Writing – original draft. CK: Data curation, Methodology, Writing – review & editing. XN: Writing – review & editing. YH: Writing – review & editing. BL: Methodology, Supervision, Writing – review & editing. GW: Writing – review & editing. JH: Writing – review & editing. HL: Conceptualization, Writing – review & editing.
